# The Relationship Between Resting Cerebral Blood Flow, Neurometabolites, Cardio-Respiratory Fitness and Aging-Related Cognitive Decline

**DOI:** 10.3389/fpsyt.2022.923076

**Published:** 2022-06-09

**Authors:** Venkatagiri Krishnamurthy, Isabella Paredes Spir, Kevin M. Mammino, Joe R. Nocera, Keith M. McGregor, Bruce A. Crosson, Lisa C. Krishnamurthy

**Affiliations:** ^1^Center for Visual and Neurocognitive Rehabilitation, Atlanta VA Healthcare System, Decatur, GA, United States; ^2^Department of Neurology, Emory University, Atlanta, GA, United States; ^3^Division of Geriatrics and Gerontology, Department of Medicine, Emory University, Atlanta, GA, United States; ^4^Department of Rehabilitation Medicine, Emory University, Atlanta, GA, United States; ^5^Department of Clinical and Diagnostic Sciences, University of Alabama at Birmingham, Birmingham, AL, United States; ^6^Birmingham/Atlanta VA GRECC, Birmingham, AL, United States; ^7^Department of Physics and Astronomy, Georgia State University, Atlanta, GA, United States; ^8^Department of Radiology and Imaging Sciences, Emory University, Atlanta, GA, United States

**Keywords:** pre-SMA, GABA, glutamate-glutamine (Glx), cerebral blood blow, MRS, aging, cardiorespirarory fitness, cognition

## Abstract

Older adults typically experience a decline in cognitive function, but improvements in physical health and lifestyle can be neuroprotective across the human lifespan. The primary objective of this study is to advance our basic understanding of how cardiorespiratory fitness and neurophysiological attributes relate to cognitive decline. While cerebral blood flow (CBF) is critical for the supply of nutrients to the tissue, the brain’s major neurotransmitters (i.e., gamma-aminobutyric acid, GABA, and glutamate-glutamine complex, Glx) are closely linked to oxidative metabolism. Within the context of flow-metabolism coupling, the critical question is how these neurophysiological parameters interplay, resulting in cognitive decline. Further, how cardiorespiratory fitness may impact aging neurophysiology and cognition is not well understood. To address these questions, we recruited 10 younger and 12 older cognitively intact participants to collect GABA and Glx using magnetic resonance spectroscopy (MRS), CBF using pseudo-continuous arterial spin labeling Magnetic Resonance Imaging (MRI), VO2max as a measure of cardiorespiratory fitness using the YMCA submax test, and cognitive and motor-cognitive measures using a battery of behavioral assessments. We observed expected differences in GABA+, Glx, and CBF between younger and older participants in pre-SMA, a frontal domain-general region. When GABA+ and Glx were related to CBF via multiple linear regression, Glx was identified as the main contributor to the model. For higher-order executive function (i.e., inhibition versus color naming), GABA*Glx*CBF interaction was critical in younger, while only Glx was involved in older participants. For unimanual motor dexterity, GABA*Glx interaction was the common denominator across both groups, but younger participants’ brain also engages CBF. In terms of selective motor inhibition, CBF from younger participants was the only major neurophysiological factor. In terms of fitness, cardiorespiratory fitness was significantly related to GABA, Glx, and motor performance when combining cohorts, but no group-specific relationships were observed. Taken together, our results indicate that Glx and CBF coupling decreases with aging, perhaps due to altered glial oxidative metabolism. Our data suggest that GABA, Glx, and CBF are engaged and weighted differently for different cognitive measures sensitized to aging, and higher fitness allows for a more efficient metabolic shift that facilitates improved performance on cognitive-motor tasks.

## Introduction

Healthy aging has been associated with alterations in mitochondrial metabolism and related loss of brain function (1). Mitochondria are the powerhouse of the cell where the bulk of oxidative metabolism occurs via chemical processes within the tricarboxylic acid (TCA) cycle and electron transport chain. Cascading from the TCA cycle is a shunt that generates glutamate (Glu), glutamine (Gln), and gamma-aminobutyric acid (GABA), brain chemicals that are often associated with neurotransmission, but also have a metabolic signature (2). Thus, aging-related changes in mitochondria could in part explain aging-related declines in glutamate and GABA (3). Animal studies show that glutamate and GABA contributions to glucose oxidation can be separated and that GABAergic neurons account for 23% of total neurotransmitter cycling and 18% of total neuronal TCA cycle (4). These findings suggest that oxidative metabolism is closely linked to changes in both glutamatergic and GABAergic neurotransmission. Further, evidence of a strong association between glutamate (Glu) neurotransmitter cycling and metabolic activity suggests that most of the metabolic activity is used to support glutamatergic signaling (5). Interestingly in the human realm, particularly in aging, a combined ^13^C/^1^H magnetic resonance spectroscopy (MRS) study showed an age-related reduction in neuronal mitochondrial metabolism and altered glial mitochondrial metabolism (6).

In the event of healthy aging, the vascular system undergoes a cascade of events that negatively affect the cerebrovascular processes, leading to decreased perfusion (7), and thereby, impaired cognition (8). While there are indications that cerebral blood flow (CBF) steadily decreases across the lifespan (9), the mechanistic underpinnings of aging-related decrease in CBF are much more complex and less understood. While well-regulated CBF is required to maintain oxygen and glucose delivery (10), increases in CBF have been reported to extend beyond the locus of oxygen utilization (11). Indeed, evidence suggests that Glu evoked influx of calcium ions in the postsynaptic neurons leads to increased vasodilatory signaling (12), suggesting that CBF is controlled by factors beyond oxidative metabolism. As mentioned above, considering that GABA and Glu are products of brain metabolism and that there is a strong flow-metabolism coupling in the brain (13), the influence of GABA and the glutamate-glutamine complex (Glx) on CBF, especially in the context of aging, is not well understood.

Combining CBF with neurotransmitter concentrations to better understand the aging brain may help to focus attention on key mechanisms, but scientific questions that combine multiple imaging modalities are not trivial. There are only a couple of reports from younger participants that assess the GABA relationship with CBF that either showed a null relationship (14), a positive relationship (15), or an inverse relationship (16). In terms of the Glx and CBF relationship, again reports do not have converging results. One report showed a positive relationship between occipital Glx and CBF (17) that does not factor in aging, and the other showed an inverse relationship between CBF and Glx measured from anterior cingulate in young to middle-aged patients with schizophrenia (18). Thus, establishing a clear aging-specific CBF and neurotransmitter (i.e., GABA+ and Glx) relationship is an understudied topic that is needed to set the platform to investigate aging-related cognitive decline.

It is well documented that cardiorespiratory fitness declines with age (19) and that age-related, longitudinal decline in cardiorespiratory fitness is non-linear (20). While there are emerging reports on how cardiorespiratory fitness impacts cerebrovascular function (i.e., CBF and cerebrovascular reactivity) in aging (7), the influence of baseline cardiorespiratory fitness on brain neurotransmitters is a nascent direction that is only now gaining prominence. For example, a recent report involving younger participants showed that repetitive transcranial magnetic stimulation (rTMS) to the pre-supplementary motor area (pre-SMA) not only increased local GABA concentration but also improved working memory at a greater rate in those with higher cardiorespiratory fitness (21). While this is exciting, such an investigation in aging that also incorporates vascular measures such as CBF is missing.

Thus, the overall objective of this study is to investigate aging-specific relationships between CBF and neurotransmitters (i.e., GABA and Glx), their role in aging-related cognitive decline, and the impact of baseline cardiorespiratory fitness on neurophysiology and cognition in aging. The specific goals of this study are: (1) to establish aging-specific relationships between CBF, GABA and Glx measured from pre-SMA, (2) to explore the impact of GABA, Glx, and CBF on domain-general cognitive and cognitive-motor functions in aging, and (3) to determine the impact of baseline cardiorespiratory fitness on neurophysiology (i.e., CBF, GABA, and Glx) and cognition within the context of aging.

## Materials and Methods

### General Procedures

Participants were included in the study if they were between 18 and 34 or 60 and 89 years of age, right-handed, native English speakers, had a Montreal Cognitive Assessment (MoCA) score of greater than 24, and without a history of depression or neurological disease. All participants provided informed consent in a process that was approved by the Emory University Institutional Review Board and Atlanta VA Research Oversight committee. All consent procedures followed the Declaration of Helsinki.

Each participant completed two study sessions: a cognitive testing session and an Magnetic Resonance Imaging (MRI) session. After quality control, 22 participants remained with good CBF maps and MR spectra. A subset of 17 participants was identified to also have physical fitness assessment data. The data is reported on the 22 and 17 participants, depending on the data type.

All MR imaging and spectroscopy data were collected on a Siemens 3T Prisma with radio frequency (RF) transmission achieved via the body coil and RF reception achieved with a 64-channel phased-array head coil. The participants were made comfortable with foam padding placed around the head and instructed not to move. The participants were presented with a white crosshair on black background for all scans except for the task-fMRI. The high-resolution T1w MPRAGE and pseudo continuous arterial spin labeling (pCASL) MRI were presented in our previous study (22). In the same session, MR spectroscopy (MRS) data was collected that is sensitized to assess localized GABA+ and glutamate-glutamine complex (Glx) concentrations (23). Full MR sequence details are provided in the pCASL and MRS acquisition sections below. In this report, we relate the MRS information with CBF, cognitive measures, and physical fitness.

### Cognitive Data Collection and Analysis

Standardized screening and behavioral assessments were administered by trained study personnel to measure cognitive and motor skills and assess lifestyle habits. Screening tools included the MoCA to ensure the recruitment of neurologically intact participants (24) and the Beck Depression Inventory-II (BDI-II) to ensure no current depressive symptoms (25). Cognitive skills were assessed by collecting the Digit Span forward and backward task to assess working memory capacity (26), the Hopkins Verbal Learning Test (HVLT-R) to assess verbal learning and short-term memory (27), the Delis-Kaplan executive function system (D-KEFS) to assess executive function (28), and verbal fluency (29). Motor skills were assessed by collecting Purdue Pegboard for manual dexterity (30), coin rotation for rapid, coordinated finger movements (31), and Halstead finger tapping to assess selective motor inhibition (32). Lifestyle habits were assessed by collecting the Godin Leisure-Time questionnaire (33, 34) to determine differences in leisure time and time spent exercising and the Sedentary Behavior questionnaire (35).

Subtest scores of the cognitive and motor assessments were analyzed separately, when appropriate. The Halstead finger tapping task using the right hand was administered in the standard fashion and the first four trials were averaged to obtain a final score. For each behavioral score, a two-sample Student’s *t*-test was administered to identify statistically significant group differences. The behavioral scores with significant differences between younger and older cohorts were then promoted to relate to neurophysiological parameters GABA+, Glx, and CBF, as well as cardiorespiratory fitness parameter VO2max.

### Pseudo Continuous Arterial Spin Labeling Magnetic Resonance Imaging Data Acquisition and Cerebral Blood Flow Quantification

The pCASL MRI acquisition and CBF quantification were described in our previous article (22). Briefly, a 2D pCASL Echo Planar Imaging (EPI) sequence was used to assess regional brain perfusion. The images were collected at the magnet isocenter to obtain an adequate blood label. The sequence parameters are as follows: FOV = 220 × 220 mm^2^, matrix = 64 × 64, TR = 4080 ms, TE = 13 ms, GRAPPA factor = 2, twenty 5 mm axial slices in ascending order with a 1 mm gap, post-labeling delay (PLD) = 1800 ms, labeling time = 1500 ms, 47 pairs of label and control acquisitions with a total scan time of 6 min 36 s. The participants were instructed to stay awake with their eyes open and be still during the duration of the scan. A fully relaxed proton density-weighted scan (M0) with similar parameters except for TR = 10 s with 2 averages were acquired to convert the perfusion signal to absolute CBF value.

The pCASL time-course was corrected for bulk-head motion and pairs of volumes were censored if the head motion was greater than 0.5 mm. The pCASL data were then spatially smoothed (Gaussian kernel FWHM = 6 mm), followed by pairwise subtraction of control and label images which were averaged to generate the mean perfusion image. The signal was converted to CBF in physiological units (mL/100 g/min) by dividing the perfusion image with the M0 image and applying a single-compartment model (36). The CBF maps were spatially transformed into MNI space.

### Magnetic Resonance Spectroscopy Acquisition and Pre-processing

The J-edited (37) MRS acquisition utilized the Center for Magnetic Resonance Research (CMRR) Spectroscopy Tools Mescher-Garwood Point Resolved Spectroscopy (MEGA-PRESS) (23) sequence to separate the small GABA+ signals from the rest of the MR spectrum (TR = 2000 ms, TE = 68 ms, voxel size = 3× 3 × 3 cm^3^, acquisition bandwidth = 2000 Hz, acquisition duration = 1024 ms, vector size = 2048, VAPOR water suppression bandwidth = 135 Hz, editing pulse bandwidth = 53 Hz, ON editing pulse = 1.9 ppm, OFF editing pulse = 7.5 ppm, total scan duration = 10 min). Each free induction decay (FID) was collected and stored separately for use in preprocessing. The CMRR Spectroscopy Tools FAST(EST)MAP (38, 39) is used to achieve a high-quality shim in the pre-SMA. The MRS voxel was left-right centered over the midline of pre-SMA on a high resolution T1w MPRAGE (sagittal 3D acquisition, TR = 2530 ms, TE = 2.96 ms, TI = 1100 ms, FA = 7°, FOV = 256 x 240 mm^2^, 176 slices, voxel size = 1× 1 × 1 mm^3^, partial Fourier = 7/8, acquisition bandwidth = 130 Hz/px, total scan duration = 8:53 min) by trained study personnel. An unsuppressed water (H_2_O) spectrum with matching acquisition parameters was also collected from the same region, except that the TR = 10 s to allow for full T_1_ relaxation.

The raw FIDs were imported into Matlab for pre-processing using in-house routines. Specifically, the FIDs were (1) corrected for phase and frequency drift (40) to improve the linewidth of the averaged spectra, (2) removal of spectra with frequency drifts greater than 10 Hz that generally occurs due to motion, (3) alignment of ON and OFF spectra at 3 ppm to reduce the occurrence of subtraction artifacts in the difference spectra, followed by (4) subtraction of averaged ON and OFF spectra. The complex time-domain data was output in.RAW format for further quantification in LCModel (41, 42).

### LCModel Fitting of Gamma-Aminobutyric Acid+ and Glutamate-Glutamine Complex in Difference Spectra

Simulated basis sets were generated using the VESPA (VErsatile Simulation, Pulses, and Analysis) (43, 44) package. The RF pulses used for the CMRR MEGA-PRESS sequence on the Siemens Prisma VE11C Syngo platform were imported into the Vespa-Pulse application and then used in the Vespa-Simulation application to simulate the full MEGA-PRESS data acquisition for both ON and OFF editing pulses. The ON/OFF spectra were then post-processed to create DIFF spectra and results output for use in the LCmodel fitting software. The following metabolites were simulated in Vespa-Simulation MEGA-PRESS: Alanine, Aspartate, Creatine, Phosphocreatine, GABA, Glucose, Glutamine, Glutamate, Glycerophosphorylcholine, Glutathione, Myo-inositol, Lactate, N-acetyl aspartate, N-acetyl aspartate glutamate, Scyllo-inositol, Taurine, Choline, Glycine. Once imported into LCModel, the DIFF spectra were modeled using the VESPA simulated basis sets in addition to a modeled baseline spline-knot spacing set at 0.6 ppm. The GABA and Glx quantifications were recorded. After LCmodel quantification, the water-scaled metabolite concentration was cerebrospinal fluid (CSF) corrected (45) based on voxel registrations and tissue segmentations performed in Gannet (46). Although it is well established that gray matter and white matter have different GABA concentrations (47), we chose to restrict our tissue correction to CSF rather than gray matter and white matter alpha-correction (45) because to the best of our knowledge, the alpha value has not been established for older participants. We show the group-level effects of CSF- versus alpha-correction in Supplementary Section 1. The parsed voxel was also transformed into MNI space via the warp matrix computed from the high-resolution T1w image for overlap across participants and localization of pre-SMA pCASL data.

### Cardiovascular Fitness Assessment

To quantify cardiovascular fitness level, participants performed a YMCA submaximal fitness test (48) on a cycle ergometer. This submaximal test was used to estimate the participant’s maximal oxygen uptake (VO2max). The YMCA test uses an extrapolation method in which heart rate workload values are obtained at 2-4 points during stages of increasing resistance and extrapolated to predict workload at the estimated maximum heart rate (e.g., 220-age). Estimated VO2max was then calculated from the predicted maximum workload and quantified in ml/kg/min using the Monark 939E Analysis software (v3.0, Vansbro, Sweden). Before beginning the test, the procedures were briefly explained, and participants completed a 2-min warm-up consisting of pedaling without load so that they could adapt to the ergometer for the first minute and then pedaling with a 0.5 kg.m load during the second minute. The YMCA submax extrapolated VO2max correlates with the measured VO2max (r = 0.86, standard error of estimate = 10%) (49).

### Statistical Models

We applied a multiple linear regression (MLR) model to test how much GABA+ (inhibitory), Glx (excitatory), and the cross-term GABA*Glx (inhibitory-excitatory interaction) may describe CBF of the pre-SMA gray matter ribbon. The model fit for:


(1) C⁢B⁢F=A+B⋅G⁢A⁢B⁢A+C⋅G⁢l⁢x+D⋅(G⁢A⁢B⁢A⋅G⁢l⁢x)+e

where CBF is the blood flow in the pre-SMA gray matter ribbon, GABA is the optimized water scaled CSF-corrected GABA+ concentration, Glx is the optimized water scaled CSF corrected Glx concentration, and A,B,C,D are parameters that are fit for during the modeling, and e is the error term.

To model the relationship between VO2max and the neurophysiological parameters GABA+, Glx, and CBF, simple linear regression was utilized, as described in Equation 2:


(2) $$N\,e\,u\,r\,o\,p\,h\,y\,s\,i\,o\,l\,o\,g\,y=A+B\cdot V\,O_{2}\,m\,a\,x+e$$

where Neurophysiology represents CSF-corrected GABA+/H2O, CSF-corrected Glx/H2O, or CBF in pre-SMA gray matter ribbon, A and B parameters are fit for during the modeling, and e is the error term.

Five models were tested to describe behavior with GABA, Glx, and CBF. Equations 3-7 show the construction of each model and the name of the model referred to in the text.

**Glx-only**


(3) b⁢e⁢h⁢a⁢v⁢i⁢o⁢r=A+B⋅G⁢l⁢x+e

**GABA-only**


(4) b⁢e⁢h⁢a⁢v⁢i⁢o⁢r=A+B⋅G⁢A⁢B⁢A+e

**GABA-by-Glx**


(5) b⁢e⁢h⁢a⁢v⁢i⁢o⁢r=A+B⋅G⁢l⁢x+C⋅G⁢A⁢B⁢A+D⋅(G⁢A⁢B⁢A⋅G⁢l⁢x)+e

**CBF-only**


(6) b⁢e⁢h⁢a⁢v⁢i⁢o⁢r=A+B⋅C⁢B⁢F+e

**GABA-by-Glx-by-CBF**


(7) b⁢e⁢h⁢a⁢v⁢i⁢o⁢r=A+B⋅G⁢l⁢x+C⋅G⁢A⁢B⁢A+D⋅C⁢B⁢F+E⋅(G⁢A⁢B⁢A⋅G⁢l⁢x)+F⋅(G⁢A⁢B⁢A⋅C⁢B⁢F)+G⋅(G⁢l⁢x⋅C⁢B⁢F)+e

The five statistical models in Equations 3–7 are ranked by their quality of fit using Adjusted R2 with criteria described in Supplementary Section 2.

All linear and multiple linear regression modeling was accomplished in JMP Pro16 using Standard Least Squares fit. The results of the whole model fit are reported with F and associated p-value. The results of each parameter fit are reported with t and associated p-value. We chose a significance threshold of *p* ≤ 0.05 for interpretation. The statistical tests were not corrected for multiple comparisons.

## Results

### Cognitive, Motor, and Lifestyle Assessments of Younger and Older Cohorts

Table 1 details the demographic information from *N* = 10 younger participants and *N* = 12 older participants, as well as cognitive, motor, and lifestyle assessments. By definition of the groups, the age is significantly different, but sex, years of education, MoCA score, and BDI total score are balanced across groups. Of the cognitive scores, the HVLT-R total recall, delayed recall, % retention, and recognition discrimination index, as well as D-KEFS inhibition versus color naming, showed aging-related differences. Of the motor cognition scores, the Purdue pegs and assembly, left hand bilateral coin rotation, and Halstead finger tapping task all show aging-related differences. The scores with significant group differences were promoted to relate to the neurophysiology and have bolded p values in Table 1.

**TABLE 1:** Summary of group-level demographic and behavioral measures.

Measure	Younger (*N* = 10)	Older (*N* = 12)	t	p
Age	23.5 ± 3.1	66.9 ± 5.0	–25.01	< 0.0001**
Sex	4F/6M	6F/6M		0.69
Years of education	15.5 ± 1.4	15.9 ± 0.9	–0.80	0.44
MOCA score	27.8 ± 1.6	27.4 ± 1.7	0.54	0.59
Beck depression inventory II	4.5 ± 6.8	5.3 ± 3.6	–0.31	0.76
**Cognitive**				
Digit span forward	10.8 ± 2.1	10.8 ± 2.2	0.05	0.96
Digit span backward	7.2 ± 1.5	6.3 ± 1.8	1.23	0.23
HVLT total recall	27.7 ± 2.5	22.3 ± 5.6	2.97	0.009**
HVLT delayed recall	10 ± 1.1	7.2 ± 3.0	3.07	0.008**
HVLT % retention	91.1 ± 8.1	77.0 ± 6.2	2.10	0.05**
HVLT recognition discrimination index	11.4 ± 0.7	10.0 ± 1.8	2.47	0.03**
D-KEFS letter fluency vs. category fluency	8.1 ± 3.3	10.3 ± 2.7	–1.63	0.12
D-KEFS category switching vs. category fluency	10.4 ± 2.4	8.8 ± 2.7	1.44	0.16
D-KEFS inhibition vs. color naming	12.1 ± 1.4	9.9 ± 2.2	2.79	0.01**
D-KEFS inhibition/switching vs. inhibition	9.7 ± 1.9	10.3 ± 1.6	–0.85	0.40
Verbal fluency FAS	39.6 ± 13.4	47.7 ± 9.1	–1.61	0.13
Verbal fluency animals & boys names	43.9 ± 5.1	43.1 ± 7.6	0.30	0.77
**Motor cognition**				
Purdue pegs (Right)	15.1 ± 1.5	11.9 ± 1.6	4.93	< 0.0001**
Purdue assembly (Right)	9.1 ± 0.9	7.5 ± 1.0	4.00	0.0007**
Coin rotation unilateral (Right)	14.7 ± 5.6	14.9 ± 3.4	–0.13	0.90
Coin rotation bilateral (Right)	13.6 ± 3.7	15.2 ± 3.6	–0.99	0.33
Coin rotation unilateral (Left)	15.3 ± 4.7	18.6 ± 3.9	–1.80	0.09*
Coin rotation bilateral (Left)	15.2 ± 3.2	18.6 ± 4.2	–2.13	0.05**
Halstead finger tapping (Right)	47.0 ± 7.4	38.4 ± 8.2	2.59	0.02**
**Lifestyle assessments**				
Godin leisure time score 1	53.6 ± 23.7	35 ± 25.1	1.57	0.14
Godin leisure time score 2	1.75 ± 1.0	2.4 ± 0.7	–1.58	0.14
Sedentary behavior questionnaire	10.6 ± 3.0	7.8 ± 3.3	1.86	0.08

*t, and p denote standard statistical parameters for t-statistics and alpha threshold, respectively.*

**Significant at p ≤ 0.05. Bolded text is to indicate significant or trending results.

### Group Comparison of Gamma-Aminobutyric Acid+, Glutamate-Glutamine Complex, and Cerebral Blood Flow in Pre-supplementary Motor Area

**FIGURE 1:**
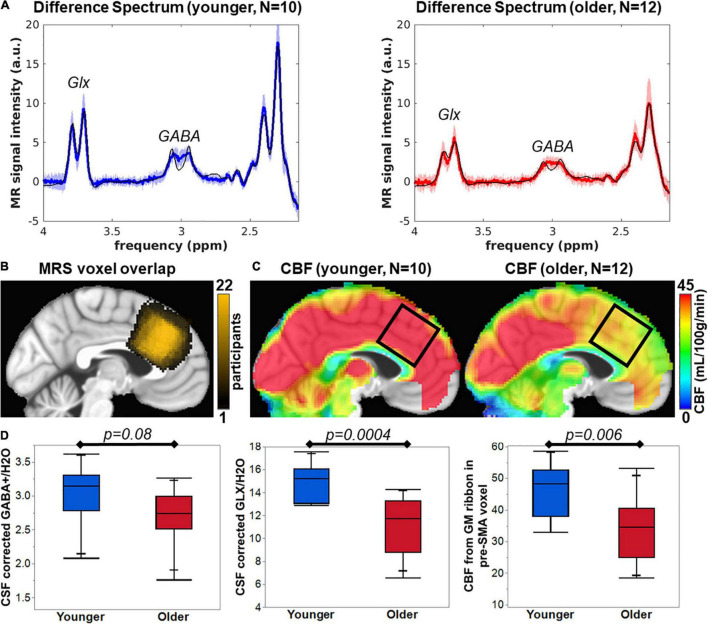
**(A)** Average difference spectra for younger (blue line) and older cohorts (red line) with shaded region representing standard deviation of the group spectra. The thin black line shows the average LCModel fit. **(B)** MRS voxel overlap in pre-SMA across *N* = 22 participants (color bar represents the overlap of number of participants). **(C)** Average CBF maps for younger and older participants with black square indicating pre-SMA region that corresponds to the MRS voxel location. **(D)** The bar plots of group comparisons for CSF corrected GABA+/H2O (institutional units), CSF corrected Glx/H2O (institutional units), and CBF from the gray matter ribbon in the MRS voxel (mL/100g/min).

Good MR spectra were defined as a total creatine (tCr) linewidth full width half maximum (FWHM) of < 18 Hz, GABA Cramer-Rao Lower Bound (CRLB) of < 8%, and Glx CRLB < 5%. Upon visual inspection, the average spectra from younger and older participants are different in peak amplitude of GABA and Glx (Figure 1A). The voxel location was generally similar across all 22 participants (Figure 1B). The range of tCr linewidth was 3.9-17.6 Hz, with all but one participant that had linewidths < 12 Hz. The younger tCr linewidth = 7.1 ± 2.3 Hz and older tCr linewidth = 9.4 ± 3.1 Hz and was not significantly different between groups (*t* = −2.0, *p* = 0.06). The range of GABA CRLB was from 3 to 7%, with younger GABA CRLB = 3.8 ± 0.63% and older GABA CRLB = 4.4 ± 1.2%. The GABA CRLB was not significantly different between cohorts (*t* = −1.57, *p* = 0.13). The Glx CRLB ranged from 2 to 4%, with younger Glx CRLB = 2.2 ± 0.4% and older Glx CRLB = 2.9 ± 0.5%. The Glx CRLB was significantly different between cohorts (*t* = −3.59, *p* = 0.002).

The voxel tissue content was determined via segmentation of the T1w anatomical image. The fractional GM content for younger = 0.51 ± 0.04 and older = 0.39 ± 0.03 was significantly different (*t* = 8.83, *p* < 0.0001). The fractional CSF content for younger = 0.12 ± 0.03 and older = 0.21 ± 0.07 was significantly different (*t* = −3.88, *p* = 0.001). The fractional WM content for younger = 0.36 ± 0.06 and older = 0.40 ± 0.08 was not statistically different (*t* = −1.07, *p* = 0.30). To account for changes in CSF content, we performed CSF correction on the GABA+ and Glx concentration. However, group differences in metabolite concentrations could still be driven by differences in GM content of the voxel and are unaccounted for in this report.

Pre-SMA CSF-corrected GABA+/H2O for younger and older participants was 3.03 ± 0.43 and 2.70 ± 0.41, respectively, which is not significantly different as tested with a two-tailed Student’s *t*-test (*t* = −1.88, *p* = 0.08, Figure 1D). CSF-corrected Glx/H2O in pre-SMA for younger and older participants was 14.94 ± 1.56 and 11.26 ± 2.44, respectively, which is significantly different as tested with a two-tailed Student’s *t*-test (*t* = −4.28, *p* = 0.0004, Figure 1D). Finally, CBF maps were computed on all participants that retained ≥ 75% of pairs for a frame-wide displacement threshold = 0.5. Upon visual inspection, the pre-SMA CBF of younger participants is higher than in older participants (Figure 1C). The CBF in the gray matter ribbon within the pre-SMA for younger and older participants was 46.45 ± 8.64 mL/100g/min and 33.93 ± 10.20 mL/100g/min, respectively, which is significantly different as tested with a two-tailed Student’s *t*-test (*t* = −3.12, *p* = 0.006, Figure 1D).

### Gamma-Aminobutyric Acid+ and Glutamate-Glutamine Complex Relationship With Cerebral Blood Flow in Whole Magnetic Resonance Spectroscopy Voxel

**FIGURE 2:**
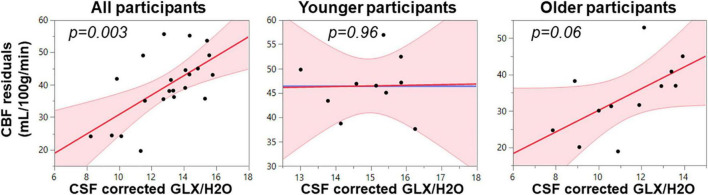
Multimodal regression plots relating CBF to Glx for all, younger, and older participants. Relationships are considered significant at *p* < 0.05.

Because GABA+, Glx, and CBF all reduce with age in pre-SMA and are representative of tissue health, we utilized multiple linear regression to assess the relationship between the neurophysiological parameters. As seen in Table 2 and Figure 2, the full model in Equation 1 describing CBF from the whole MRS voxel is significant for all participants (*F* = 6.47, *p* = 0.004) with Glx as the significant parameter (*t* = 3.46, *p* = 0.003). The Older participants drive the relationship, where the Glx parameter significantly describes CBF in the older group (*t* = 2.14, *p* = 0.06), but not in the younger group (*t* = 0.06, *p* = 0.96). The GABA and the GABA*Glx cross-term are not significant for either group or across all participants.

**TABLE 2:** Multiple linear regression results to model cerebral blood flow (CBF) with gamma-aminobutyric acid (GABA), glutamate-glutamine complex (Glx), and GABA-by-Glx cross term for all, younger, and older participants.

		Whole model		Individual parameter			
		F	p	parameter	β	t	p
All	CBF	**6.47**	0.004*	GABA	0.18	0.03	0.97
				**Glx**	**2.99**	**3.46**	0.003*
				GABA⋅Glx	1.82	1.55	0.14
Younger	CBF	2.39	0.17	GABA	14.61	1.59	0.16
				Glx	0.13	0.06	0.96
				GABA⋅Glx	0.19	0.04	0.97
Older	CBF	1.70	0.24	GABA	−11.67	−1.33	0.22
				**Glx**	**2.98**	**2.14**	**0.06**
				GABA⋅Glx	0.22	0.09	0.93

*β denotes the model coefficient, and F, t, and p denote standard statistical parameters for F-statistics, t-statistics and alpha threshold, respectively.*

*Significant at p ≤ 0.05. Bolded text is to indicate significant or trending results.

### Influence of VO2max on Gamma-Aminobutyric Acid+, Glutamate-Glutamine Complex, and Cerebral Blood Flow

VO2max measured in mL/kg/min is a measure of cardiorespiratory fitness. In a subset of participants of 7 younger and 10 older, the VO2max was significantly different between groups (*t* = 4.13, *p* = 0.001), where younger participants had an average VO2max of 41.9 ± 7.5 mL/kg/min and older had an average VO2 max of 26.9 ± 7.1 mL/kg/min.

**FIGURE 3:**
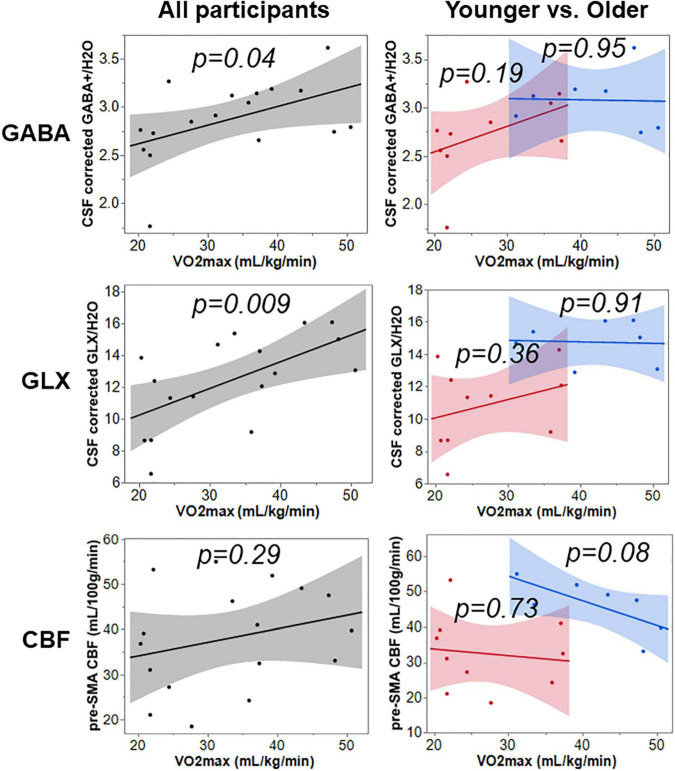
The relationship of VO2max to GABA+, Glx, and CBF across all, younger, and older participants.

Simple linear regression was used to test if VO2max separately predicted neurophysiological measures GABA+, Glx, and CBF using Equation 2. The results for all, younger, and older participants are summarized in Figure 3 and Table 3. The overall regression for GABA+ and Glx in all participants was statistically significant, but VO2max did not predict pre-SMA gray matter CBF in all participants.

**TABLE 3:** Model relating VO2max to neurophysiological measures gamma-aminobutyric acid+ (GABA+), glutamate-glutamine complex (Glx), and cerebral blood flow (CBF).

	β	R^2^	F	p
**All participants**				
**GABA+**	**12.63**	**0.24**	**4.85**	0.04**
**Glx**	**2.25**	**0.38**	**9.10**	0.009**
CBF	0.25	0.07	1.21	0.29
**Younger participants**				
GABA+	−0.79	0	0	0.95
Glx	−0.31	0	0.01	0.91
**CBF**	−**0.71**	**0.50**	**4.92**	**0.08**
**Older participants**				
GABA+	7.57	0.20	2.00	0.19
Glx	0.93	0.10	0.94	0.36
CBF	−0.09	0.02	0.13	0.73

*β denotes the model coefficient, and F, R^2^, and p denote standard statistical parameters for F-statistics, proportion of the variance and alpha threshold, respectively.*

**Significant at p ≤ 0.05. Bolded text is to indicate significant or trending results.

### Modeling Cognitive Measures With Gamma-Aminobutyric Acid+, Glutamate-Glutamine Complex, and Cerebral Blood Flow

We tested five statistical models (Equations 3–7) to describe the behavioral measures from Table 1 and ranked the quality of the fit using Adjusted R^2^. Only those behavioral measures that showed significant differences between younger and older participants were promoted to the modeling phase as the goal of this study is to investigate the physiological underpinnings of cognitive decline. The comprehensive tables of Adjusted R^2^ for all, younger, and older participants can be found in Supplementary Section 2. Across all participants, the behavioral measures best fit by one of the five statistical models is summarized in Table 4. Although HVLT-R subtests, right hand Purdue pegs, and left hand bilateral coin rotation showed significant differences between younger and older task performance, the modeling itself did not show results of interest. The final behaviors that were significantly described by the neurophysiology were D-KEFS inhibition vs. color naming, right hand Purdue assembly, and right hand Halstead finger tapping task.

**TABLE 4:** Best fit neurophysiological model for subtests of D-KEFS, Purdue, and Halstead.

	Best fit model	R^2^	Adj R^2^	F	p	Parameter	β	t	p
**All**									
D-KEFS inhibition vs. color naming	**Glx-only**	**0.43**	**0.40**	**15.27**	0.0009*	**Glx**	**0.51**	**3.91**	0.0009*
Purdue assembly (Right)	GABA-by-Glx-by-CBF	0.49	0.29	2.44	0.08	GABA	−0.40	−0.52	0.61
						Glx	0.17	1.07	0.30
						CBF	0.02	0.63	0.54
						GABA⋅Glx	−0.13	−0.49	0.63
						GABA⋅CBF	0.04	0.40	0.69
						Glx⋅CBF	−0.02	−1.49	0.16
Halstead finger tapping (Right)	**Glx-only**	**0.26**	**0.22**	**6.87**	0.02*	**Glx**	**1.62**	**2.62**	0.02*
**Younger**									
D-KEFS inhibition vs. color naming	**GABA-by-Glx-by-CBF**	**0.96**	**0.89**	**13.78**	0.03*	GABA	1.15	1.38	0.26
						Glx	0.19	1.10	0.35
						**CBF**	**0.19**	**6.34**	0.008*
						GABA⋅Glx	−0.86	−1.28	0.29
						**GABA⋅CBF**	**0.67**	**6.29**	0.008*
						**Glx⋅CBF**	−**0.09**	−**3.06**	0.05*
Purdue assembly (Right)	GABA-by-Glx	0.63	0.44	3.37	0.10	**GABA**	−**2.39**	−**2.79**	0.03*
						Glx	0.22	1.06	0.33
						GABA⋅Glx	−0.73	−1.76	0.13
Halstead finger tapping (Right)	CBF-only	0.34	0.26	4.09	0.08	**CBF**	−**0.50**	−**2.02**	**0.08**
**Older**									
D-KEFS inhibition vs. color naming	Glx-only	0.29	0.22	4.02	0.07	**Glx**	**0.48**	**2.01**	**0.07**
Purdue assembly (Right)	GABA-by-Glx-by-CBF	0.82	0.60	3.74	0.08	GABA	0.38	0.54	0.61
						Glx	0.02	0.18	0.87
						**CBF**	**0.06**	**2.37**	**0.06**
						GABA⋅Glx	0.02	0.08	0.94
						GABA⋅CBF	−0.10	−0.90	0.41
						**Glx⋅CBF**	−**0.05**	−**3.06**	0.03*
Halstead finger tapping (Right)	None	−	−	−	−	−	−	−	−

*β denotes the model coefficient, and F, t, R^2^, Adj. R^2^ and p denote standard statistical parameters for F-statistics, t-statistics, proportion of the variance, proportion of variance adjusted for number of predictors in the model and alpha threshold, respectively.*

*Significant at p ≤ 0.05. Bolded text is to indicate significant or trending results.

### Modeling Cognitive Measures With VO2max in Combination With Gamma-Aminobutyric Acid+, Glutamate-Glutamine Complex, or Cerebral Blood Flow

We chose to test if the behavior could be described by cardiorespiratory fitness level as measured by VO2max. As seen in Table 5, VO2max relates significantly with right hand Purdue Assembly and right hand Halstead finger tapping task, but these relationships do not hold in the younger or older cohorts. The significant models relating neurophysiology with cognitive measures described above were further graduated to investigate if physical fitness via VO2max could describe any additional variance in behavior. However, when VO2max was combined with GABA, Glx, or CBF to model behavior, significance was not reached.

**TABLE 5:** Linear regression of behavioral measures with VO2max.

	β	R^2^	F	p
**All**				
D-KEFS inhibition vs. color naming	0.05	0.06	0.88	0.36
Purdue assembly (Right)	**0.07**	**0.32**	**7.26**	0.02**
Halstead finger tapping (Right)	**0.44**	**0.21**	**4.09**	**0.06**
**Younger**				
D-KEFS inhibition vs. color naming	−0.11	0.31	2.26	0.19
Purdue assembly (Right)	0.01	0	0.01	0.91
Halstead finger tapping (Right)	0.47	0.19	1.18	0.33
**Older**				
D-KEFS inhibition vs. color naming	< 0.00	0	0	0.99
Purdue assembly (Right)	0.03	0.04	0.34	0.57
Halstead finger tapping (Right)	−0.32	0.08	0.73	0.42

*β denotes the model coefficient, and F, R^2^, and p denote standard statistical parameters for F-statistics, proportion of the variance and alpha threshold, respectively.*

**Significant at p ≤ 0.05. Bolded text is to indicate significant or trending results.

## Discussion

Cognitive aging is a natural process wherein older adults typically experience a decline in many cognitive functions that can negatively impact their quality of life. Current trends in brain research indicate that improvements in physical health and lifestyle approaches can be neuroprotective across the human lifespan. From this viewpoint, the primary objective of this study was to advance our basic understanding of how baseline cardiorespiratory fitness along with aging brain physiology impact the cognitive decline such that refined cognitive rehabilitations programs can be built to improve the quality of life in older persons with cognitive disorders. As pre-SMA is a critical node in cognitive control, we designed our experiments to explore the above scientific question in this brain area. Finally, considering the preliminary nature of this study, we found some interesting results that are discussed in the following sub-sections along with limitations and suggestions for future studies.

### Aging-Related Differences in Pre-supplementary Motor Area Gamma-Aminobutyric Acid+, Glutamate-Glutamine Complex, and Cerebral Blood Flow

We identified significant group differences in Glx and CBF. However, in this cohort, the GABA+ concentration is not significantly different between younger and older participants. This was surprising, as other literature [for example see Simmonite et al. (50)] instead shows that GABA+ exhibits a greater aging-related difference than Glx, but it is unknown why the sensitivity in our data favors Glx. Perhaps analysis differences, including the use of different software tools that model the GABA signal differently (for example LCModel versus Gannet) or the use of normalizing neurometabolites (for example Cr versus H2O) could cause differences in study outcomes. Or it could be that pre-SMA is a heavily glutamatergic region and may not show a large reduction in GABA+ due to aging-related processes, or that not all brain regions may show similarly strong reductions in GABA+ across the lifespan or with age (51). We chose to perform CSF-tissue correction for GABA instead of accounting for GABA differences in gray matter and white matter via alpha-correction for two reasons: (i) to the best of our knowledge, the alpha value has not been established in older persons and (ii) the CSF- and alpha-corrected GABA+/H2O values were highly correlated in both younger and older cohorts (see Supplementary Section 1). Thus, all relationships established using conservatively chosen CSF-corrected GABA+/H2O in this study should translate well to alpha-corrected GABA values that may be undertaken in future studies. It should be noted that the GM content of the voxel was significantly greater in the younger cohort compared to the older cohort, which is unaccounted for and may be the driver of group differences. It is currently unknown if the GABA and Glx content per cell is reduced with age or if the amount of neurotransmitter per cell is preserved and simply the number of cells is reduced with age. It is likely an interaction of these two processes, as the aging-related reduction in brain metabolism (and metabolite production) is a precursor to programmed cell death and eventual reductions in GM and WM (52). More work is needed to understand how to correct for GM differences in aging and disease to accurately quantify MRS-based GABA and Glx concentrations.

### Cerebral Blood Flow, Gamma-Aminobutyric Acid+ and Glutamate-Glutamine Complex Relationships in an Aging Model

Blood is the mode by which all tissues receive metabolic substrates such as glucose and oxygen, where blood flow to a region can be increased in times of high metabolic demand (i.e., functional hyperemia). Thus, modeling CBF using GABA+ and Glx was a means of understanding the degree of influence each of these neurometabolites has on pre-SMA blood flow. This is especially interesting in the context of aging-related changes, as we have identified the expected group differences in all three parameters being entered into the model. Below we detail the findings of modeling CBF with GABA+ and Glx using multiple linear regression.

Currently, the literature is conflicted in terms of the relationship between GABA+ and CBF. In this study, we did not find any significant aging-specific relationships between GABA+ and CBF measured from pre-SMA when entered into a multilinear regression model. Muthukumaraswamy et al. (14) is another study that did not find a relationship between GABA+ and CBF. While we utilized a pCASL sequence in pre-SMA, (14) used a pulsed ASL sequence in primary visual areas, indicating that different ASL implementations and different brain regions may not be the source of discrepancy. On the other hand, Donahue et al. showed conflicting relationships between occipital GABA+ and CBF in younger participants using two different approaches: (i) positive GABA and CBF relationship when analyzing the OFF period from a visual task-CBF paradigm that incorporated a pulsed ASL sequence (15), and (ii) inverse GABA and CBF relationship when analyzing a multi-PLD pCASL sequence to account for the variability in arterial arrival time (AAT) (16). Thus, in their later article where they used the pCASL sequence with multiple PLD (16), it is remarkable to note that accounting for AAT variability renders significant inverse relationships with GABA+ which is opposite to their earlier finding. While it is valuable information to account for inter-subject variability in AAT, multi-PLD ASL measurements also come at the cost of very long scan duration which is not favorable for clinical translation. Further, multi-PLD approach does not accurately capture the CBF especially at lower PLD values as the labeled blood remains in the macrovasculature. The discrepancies in their findings could also stem from the 2010 work which extracted CBF measures from the OFF period of a boxcar visual task-CBF paradigm (15), which is not equivalent to pure baseline/resting CBF measurements. Secondly, pulsed ASL can render lower SNR in older participants, and finally their ASL acquisition approach would not allow for quantification of absolute CBF values. Thus, more work is necessary to identify the relationship between GABA+ and CBF, especially considering the hypothetical basis for such a relationship discussed next.

Considering that GABAergic neurons account for 23% of total neurotransmitter cycling and 18% of the total neuronal tricarboxylic acid (TCA) cycle (4), and that flow and metabolism have a linear relationship (13), we expected an age-relevant positive relationship between GABA+ and CBF. However, in light of the null relationship observed between GABA+ and CBF in both younger and older groups, we propose a few plausible factors: (a) a large variability in CBF and oxygen metabolism due to differences in brain state (53) may preclude the detection of a GABA and CBF relationship, (b) the inherent neural and vascular properties of pre-SMA may differ from primary sensory brain areas such as visual cortex, or (c) in the resting condition, a higher rate of TCA cycling due to more CBF does not necessarily indicate that more intermediates such as GABA are generated (54). While multiple-linear regression (MLR) is appropriate to jointly model the influence of GABA+, Glx and their interaction on CBF - which yielded a null relationship between GABA+ and CBF, our unpublished data where we incorporated simple linear regression between GABA+ and CBF resulted in a significant positive relationship in younger participants s. This result could suggest that for these specific measures, we may not have had enough power for robust MLR modeling and something worthwhile to explore in future studies.

In terms of Glx and CBF, we observe a significant positive relationship when both groups are combined. It is interesting to note that our results are consistent with previous reports (18, 55) that focused on brain areas different from pre-SMA. Note that adenosine triphosphate (ATP), the energy cache of the brain, is generated from glycolysis and mitochondrial oxidative phosphorylation in both neurons and glia (56), which is tightly coupled to the arterial vascular system (56, 57). More specifically, the glutamate-glutamine cycling which is housed in the astrocytes has been shown to decrease by ∼28% in older participants and is related to reductions in glial mitochondrial metabolism of elderly participants compared to young (6). Considering that cerebrovascular properties (i.e., endothelial functioning) also deteriorate due to aging, the lack of a relationship in older might be due to aging-related altered metabolic and vascular functions that likely requires flow-metabolism coupling to maintain homeostasis and meet energy combustion demands.

### Cerebral Blood Flow, Gamma-Aminobutyric Acid+, and Glutamate-Glutamine Complex Measured From Pre-supplementary Motor Area –Its Role in Aging-Related Cognitive Decline

In addition to other frontal (for example, dorsolateral prefrontal cortex, inferior frontal gyrus) and sub-cortical (for example, basal ganglia) brain areas, pre-SMA is considered to be a critical brain area in the cognitive control of actions that require rapid updating, inhibition, or switching, as well as working memory (58). Furthermore, the pre-SMA and SMA have cytoarchitectural gradients (59) that possibly may also support a gradient in cognitive functions wherein more anterior pre-SMA may potentially support higher-order cognitive functions (such as executive function, attention, working memory, etc.) and the more posterior pre-SMA that is closer to proper SMA may support cognitive-motor functions. While at this point the proposed (anterior to posterior) gradient in cognitive functions along pre-SMA is purely speculative, we are the first to attempt how GABA+, Glx, and CBF govern various cognitive and motor-cognitive measures, especially in the context of aging.

While we collected an array of cognitive measures, note from Tables 1, 4 that we only promoted those measures that showed a significant aging-related difference for MLR modeling (with GABA+, Glx, and CBF). This was done to truly understand the neurophysiological underpinning of aging-related cognitive decline. The ones that turned out to be significantly described by GABA+, Glx, and CBF were D-KEFS inhibition versus color naming, right hand Purdue Assembly, and right hand Halstead finger tapping measures. While GABA+, Glx, and CBF are interrelated, it was intriguing to note how the influence of each of those various physiological measures either by themselves or in some combination with other physiological measures explained more variance in each of those different cognitive measures. For D-KEFS inhibition versus color naming, in older, only Glx was significant which suggests that altered glutamine cycling that results in altered glial metabolism (6) might influence the decline in executive function. In younger, we observed that GABA-by-Glx-by-CBF interaction is critical for higher-order executive functioning which also suggests that lack of Glx interaction with GABA and CBF might be another contributing factor for poorer performance in older participants. In terms of manual dexterity (measured using Purdue assessment), considering that we only assessed for right-hand dexterity from right-hand dominant participants, it is interesting to note that GABA-by-Glx interaction is preserved across aging, but GABA-by-Glx interaction with CBF was observed in younger which was not observed in older. For selective motor inhibition (measured using Halstead-Reitan finger tapping assessment), we note that CBF from younger provided a significant relationship. Intriguingly, neither GABA+ nor Glx governs this motor task, and further, in older participants, decreased CBF might be the potential cause for selective motor inhibition failures. Tying all this back to the cognitive gradient across pre-SMA (i.e., anterior to posterior), we note that in the posterior pre-SMA which is closer to motor-dominant functional brain areas, CBF primarily governs cognitive-motor functions. On the other hand, in anterior pre-SMA, which is closer to areas that govern higher-order cognitive functions, we observe greater involvement of GABA and Glx interactions. While this is a very preliminary and cursory observation, it is still an interesting finding that is worth a deeper investigation.

### Impact of Cardiorespiratory Fitness on Neurophysiology and Cognition in an Aging Model

In recent years, research shows that cerebrovascular control and its integration with other physiological systems (such as cardiovascular and pulmonary) play a key role in improved brain functioning via rehabilitation programs (60). Aerobic exercise is an important rehabilitation approach that can non-pharmacologically invoke cardiovascular responses that improve cerebrovascular function (61) (for example, CBF), and neurophysiology (62, 63) (i.e., GABA and glutamate). However, the impact of baseline cardiorespiratory fitness (which is an index of sedentariness) on cerebrovascular and neurophysiology is not well characterized. This is a gap that must be filled to understand exercise-induced improvements in brain function and cognition. While this line of research is gaining traction, recent reports have started to show that baseline cardiorespiratory fitness influences rTMS-induced cognitive improvements and changes in GABA (21) and cerebrovascular physiology (7). In our study, we found an aging-related decrease in VO2max in older participants but did not find differences in the self-reported sedentariness between groups (Table 1). While we observed that VO2max relates positively with GABA+ and Glx when both groups were combined (see Figure 3), we did not find any significant relationships when only considering younger and older cohorts separately. Another study in postmenopausal women (age = 59 ± 3 years) also did not find any relationship between VO2max and GABA+ from the motor cortex (64). From our previous section, we note that Glx plays a critical role in brain functioning and cognition in older participants. Considering that along with the VO2max being lower in older participants, we expected a positive relationship between baseline VO2max and Glx. The observed null relationship in younger and older groups could be due to a small subject pool or an inherent property specific to pre-SMA, which needs to be explored further in future studies. However, at the combined subject level, we do observe a significant positive relationship between VO2max and both GABA+ and Glx suggesting that fitness level does influence metabolic shift that is characterized by increased reserve of neurotransmitters *via* nonoxidative metabolism of carbohydrate substrates (62).

In terms of CBF, we did not find a significant relationship with VO2max when all subjects were combined or in the older group. We did, however, observe an inverse relationship between VO2max and CBF in younger, which suggests that increased fitness level requires lower resting CBF to meet the regional metabolic demands. While this is intuitive, this observation does not corroborate the Intzandt et al. (65) study (7), where they observed an inverse relationship in older participants. Again, considering that we earlier showed that Glx and CBF are highly coupled in the older and noting that older participants were less fit in our study, we expect a positive relationship between resting VO2max and CBF. One keynote is that the above-mentioned difference between our study and Intzandt et.al (7) could be stemming from the fact that we used an estimated VO2max while Intzandt et al measured VO2peak. While VO2max is more comparable across various physical exercise modalities (for example, aerobic exercise versus dance versus walking), VO2peak can vary and thereby lead to ambiguous interpretations. Additionally, Intzandt et.al utilized a dual-echo simultaneous BOLD-ASL during hypercapnia with a PLD of 900ms which is sub-optimal for CBF quantification as the blood would still be in the macrovascular compartment.

In terms of VO2max predicting cognitive measures, we found that right hand unilateral Purdue assembly and right hand unilateral Halstead finger tapping was significantly predicted by VO2max when all subjects were combined. Note that at the combined group level, VO2max is also related to GABA+ and Glx suggesting that higher fitness allows for a more efficient metabolic shift that facilitates greater motor performance (i.e., dexterity and selective motor inhibition). Within each group, we did not find any significant results when interaction terms were omitted from MLR modeling. While investigating how the interaction between CBF, neurotransmitters, and VO2max describes cognitive measures is very interesting, unfortunately, our data did not allow us to accomplish that at the individual group level as we would be fitting for more number of parameters than available participants. While it is exciting to learn from the current study that baseline cardiorespiratory fitness impacts GABA+, Glx and behavior separately, future work on larger cohorts should consider using advanced modeling to investigate how cognition is governed by the complex interplay between cardiorespiratory and neurophysiological measures.

### Limitations and Future Directions

Considering the nature of scientific questions undertaken in this study, it not only required multi-pronged measurements (i.e., GABA+, Glx, CBF, VO2max, and behavior), but also the challenge of combining such various measurements. Considering that, indeed we expected and encountered several limitations. In terms of ASL, we implemented a PLD of 1800 ms which may have underestimated the CBF in the older group exhibiting longer arterial transit times. While we based our ASL sequence optimization as outlined in the ASL white paper (36), future work should increase the PLD safely to ≥ 2000 ms to better account for aging-related delays in arterial transit time while still maintaining the SNR of the difference signal.

We quantified metabolites GABA+ and Glx from the difference MR spectrum. Our quantification of GABA is designated “GABA+” because macromolecules are coedited with the metabolite of interest. The coedited macromolecule pool likely adds additional variance to the cohort level measurement of GABA+ (66) and is a limitation of this study because group level differences could be driven by macromolecule content rather than the metabolites of interest. Future studies should attempt to measure a non-macromolecule contaminated GABA spectrum to improve understanding of the relationship with peripheral physiology, behavior, and neurometabolites (67). Another limitation of this study is that we quantified Glx from the difference edited spectrum instead of acquiring a separate short echo PRESS. The Glx signal is co-edited with GABA+ when applying an editing pulse at 1.9 ppm, but the acquisition parameters are not optimized for Glx. In effect, other studies have shown that the stability of Glx quantification is improved when collected using a short echo PRESS as compared to the difference spectrum (68). We chose to quantify Glx from the difference spectrum instead of collecting a separate scan for two reasons: (1) the additional scan time of 4-5 min (including water calibration) would preclude this protocol from collecting other multi-modal data to describe aging-related brain changes and (2) an expert panel on edited MRS is in consensus that co-edited metabolites that are not overlapping (as is the case with Glx) can be “quantified through spectral fitting using appropriately constructed basis sets” (69) and can be done with our dataset due to the quality of the fit (our fit provides a Glx CRLB of 4% or less) (70). We have constructed tailored basis sets for this study using VESPA such that our Glx data is representative of expected aging-related differences in pre-SMA. However, because Glx is a combination of glutamate and glutamine, and glutamine is not a neurotransmitter, it may be beneficial to quantify glutamate and glutamine separately to better understand the relationship with peripheral physiology and behavior. It has been shown that glutamate and glutamine can be resolved independently from a GABA MEGA-PRESS acquisition at 3T using the QUEST software (70), but it is not known if LCModel has similar sensitivity. Thus, we quantify GABA+ and Glx to be conservative in our analytic approach using LCModel, but future studies should explore if GABA (non-macromolecule contaminated) and glutamate are able to improve the prediction of the physiology and behavior.

As can be seen from our methods section, we collected a relatively comprehensive battery of cognitive measures, but unfortunately, a lot of them did not yield aging-related differences either due to noise in the behavioral measurements and/or due to missing and incomplete data (especially in older) potentially due to mental fatigue from the long behavioral session. Although some behavioral tests showed significant relationships with the brain physiology, we did not correct for multiple comparisons because the results would not survive this correction and must therefore be interpreted with caution. This issue is further compounded by the small sample size and requires further work with a larger cohort, ideally with a lifespan study encompassing younger, midlife, and older participants. While it is satisfying that our comprehensive investigation from this study allowed us to identify key measures to study the physiological underpinnings of cognitive decline, more work is necessary to develop aging-sensitive cognitive assessments.

While the YMCA test has the advantage of being reliable and safer to administer than a max VO2 test, it does present with a few limitations. First, the extrapolated output can be influenced by the variability in maximum heart rate in individuals. As such, it may underestimate the fitness of those with a high maximum heart rate, and overestimate fitness with advancing age. Additionally, because it is performed on a cycle ergometer it could favor those with a history of cycling. Finally, the sample size was another limitation for this multimodal study as datasets that survive the QC threshold across all modalities can significantly shrink the effective and usable sample size for analysis.

## Conclusion

Cerebral blood flow and the brain’s major neurotransmitters GABA and Glx are physiological parameters that can be directly modulated via interventions. Thus, investigating the impact of these neurophysiological factors (either independently or jointly) on cognitive functions is valuable for advancing neurocognitive rehabilitation approaches. From that standpoint, this study shows that GABA, Glx, and CBF play a distinct role in aging-specific changes in executive function and motor performance (i.e., dexterity and selective motor inhibition). Further, we show that resting cardiorespiratory fitness does relate to brain health and cognition suggesting that aerobic exercise interventions are very beneficial in aging and aging-related neurodegenerative population.

## Data Availability Statement

The raw data supporting the conclusions of this article will be made available by the authors, without undue reservation.

## Ethics Statement

The studies involving human participants were reviewed and approved by the Emory University Institutional Review Board and Atlanta VA Research Oversight committee. The patients/participants provided their written informed consent to participate in this study.

## Author Contributions

VK and LK: conceptualization of the study and MR acquisition design. VK: MRI analysis design. LK: MRS analysis design. VK, LK, and IP: MRI and MRS analysis. JN: cardiorespiratory design. BC: cognitive behavior design. KMc: cognitive motor design. KMa: cardiorespiratory data collection and analysis. LK and IP: statistical analysis. VK, LK, JN, KMc, and BC: data collection. VK, LK, IP, and JN: manuscript writing. VK, LK, and BC: manuscript editing. All authors contributed to the article and approved the submitted version.

## Conflict of Interest

The authors declare that the research was conducted in the absence of any commercial or financial relationships that could be construed as a potential conflict of interest.

## Publisher’s Note

All claims expressed in this article are solely those of the authors and do not necessarily represent those of their affiliated organizations, or those of the publisher, the editors and the reviewers. Any product that may be evaluated in this article, or claim that may be made by its manufacturer, is not guaranteed or endorsed by the publisher.
