# Supplementary material for: The Relationship Between Resting Cerebral Blood Flow, Neurometabolites, Cardio-Respiratory Fitness and Aging-Related Cognitive Decline
Source: Front Psychiatry. 2022 Jun 9;13:923076. doi: 10.3389/fpsyt.2022.923076 (PMC9218954; doi:10.3389/fpsyt.2022.923076)
Supplement: Supplementary file 1 [file Data_Sheet_1.docx]

Supplementary information for:

**The relationship between resting cerebral blood flow, neurometabolites, cardio-respiratory fitness, and aging-related cognitive decline**

Venkatagiri Krishnamurthy^1,2,3^*, Isabella Paredes Spir^1^, Kevin M. Mammino^1^, Joe R. Nocera^1,2,4^, Keith M. McGregor^1,5^, Bruce A. Crosson^1,2^, Lisa C. Krishnamurthy^1,6,7^

^1^Center for Visual and Neurocognitive Rehabilitation, Atlanta VA Healthcare System, Decatur, GA, United States,

^2^Dept. of Neurology, Emory University, Atlanta, GA, United States,

^3^Dept. of Medicine, Division of Geriatrics and Gerontology, Emory University, Atlanta, GA, United States,

^4^Dept. of Rehabilitation Medicine, Emory University, Atlanta, GA, United States

^5^Dept. of Clinical and Diagnostic Sciences, University of Alabama at Birmingham, Birmingham, AL, United States

^6^Dept. of Physics & Astronomy, Georgia State University, Atlanta, GA, United States,

^7^Dept. of Radiology and Imaging Sciences, Emory University, Atlanta, GA

*Supplementary section 1: Comparison of CSF and alpha-correction of GABA+/H2O concentration*


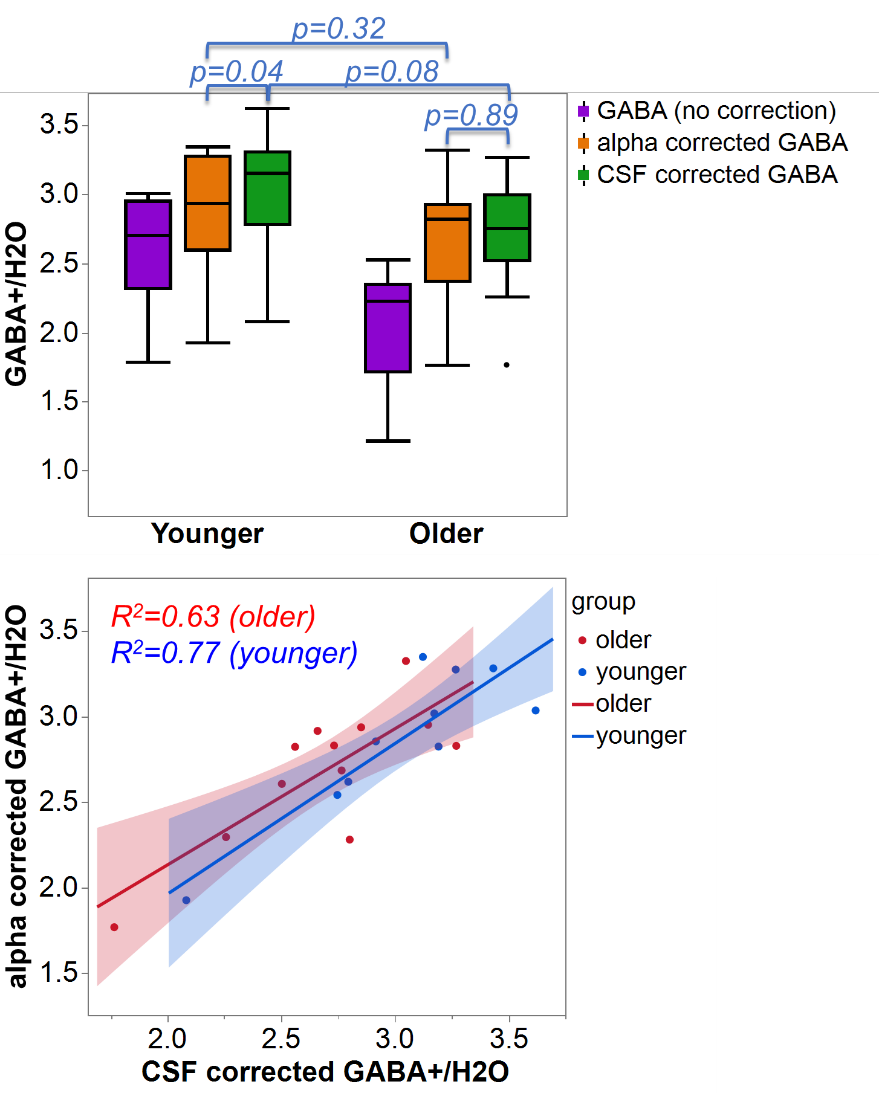


**Supplementary Figure 1: (Top panel)** Box plots of the uncorrected, alpha corrected, and CSF corrected GABA+/H2O concentration in institutional units for younger and older cohorts. The p values indicate the statistical significance in either a paired ttest (within group, across methodologies) or ttest (across groups, within methodology). **(Bottom panel)** Correlation between alpha-corrected and CSF-corrected GABA+/H2O in younger (blue) and older (red) groups.

Although it is well established that grey matter and white matter have different GABA concentrations (Mikkelsen et al., 2016), we chose to restrict our tissue correction to CSF rather than grey matter and white matter alpha-correction (Harris et al., 2015) because to the best of our knowledge, the alpha value has not been established for older participants. Here we show the effects of performing CSF correction and alpha correction of the GABA+/H2O concentration.

As seen in **Supplementary Figure 1**, the uncorrected GABA+/H2O concentration is different between the younger and older cohorts (t=3.2, p=0.005). After tissue correction (both CSF- or alpha-correction) there were no differences between the younger and older cohorts, although the differences were smaller in the alpha-corrected values. The alpha-correction is significantly different than the CSF-correction in younger participants (t=2.42, p=0.04), but not older participants (t=0.14, p=0.89). Thus, correcting for alpha adjusted grey matter and white matter GABA content has an effect in younger participants, but not in older. This could be perhaps because the alpha value changes with age, and specifically optimal alpha value in aging needs to be investigated thoroughly in future studies. Finally, the CSF- and alpha-corrected GABA+/H2O concentrations are highly correlated (R^2^=0.77, F(1,8)=26.66, p=0.0009 for younger participants and R^2^=0.63, F(1,10)=17.06, p=0.002 for older participants). Due to these corroborating linear relationships between the methodologies, it seems the difference in correction methodology amounts to a scaling factor in this cohort, such that relationships identified between CSF-corrected GABA and peripheral physiology or behavior are likely to remain the same even if alpha-correction were used.

Thus, because the alpha value is not established in older participants, and because the CSF- and alpha-corrected GABA+/H2O are highly correlated, we chose to use CSF-corrected GABA+/H2O.

*Supplementary section 2: Identifying a neurophysiological model to describe behavior*

The behavior detailed in **Table 1** was further promoted to be modeled with neurophysiological parameters GABA, GLX, and CBF using 5 models described in **Equations 3-7**. The model that best fit the behavioral data was chosen via the following criteria:

1. The ANOVA of the model must reach significance at p<0.05 or be trending at 0.05<p<0.10.
2. Within the significant/trending models, the adjusted R^2^ must be maximum.

The adjusted R^2^ will always be lower than the R^2^, as the adjusted R^2^ takes into consideration the addition of modeling parameters. Due to this feature, the adjusted R^2^ can be compared across models with different number of parameters to identify the most robust model that describes behavior. **The Supplementary Table 1** summarizes the adjusted R^2^ for each model and indicates with Asterix whether the ANOVA reached trending (*) or significance (**). The final chosen model is highlighted in yellow within **Supplementary Table 1**.

**Supplementary Table 1:** Adjusted R^2^ for models GLX-only, GABA-only, GABA-by-GLX, CBF-only, and GABA-by-GLX-by-CBF.

|  | **Glx only** | **GABA only** | **GABAxGLX** | **CBF only** | **GABAxGLXxCBF** |
| --- | --- | --- | --- | --- | --- |
| **All participants** |  |  |  |  |  |
| DKEFS inhibition vs color naming | **0.40**** | 0.08 | **0.35**** | **0.24**** | **0.28*** |
| Purdue Assembly (Right) | **0.25**** | -0.02 | **0.28**** | **0.15**** | **0.29*** |
| Halstead Finger Tapping (Right) | **0.22**** | -0.01 | 0.16 | -0.04 | 0.24 |
| **Younger participants** |  |  |  |  |  |
| DKEFS inhibition vs color naming | 0.02 | 0.07 | -0.18 | **0.33**** | **0.89**** |
| Purdue Assembly (Right) | 0.04 | **0.36**** | **0.44*** | 0.16 | 0.08 |
| Halstead Finger Tapping (Right) | -0.1 | -0.09 | -0.23 | **0.26*** | 0.47 |
| **Older participants** |  |  |  |  |  |
| DKEFS inhibition vs color naming | **0.22*** | -0.09 | 0.1 | -0.06 | 0.16 |
| Purdue Assembly (Right) | 0.07 | -0.03 | -0.13 | 0.15 | **0.60*** |
| Halstead Finger Tapping (Right) | 0.02 | -0.07 | -0.18 | -0.1 | -0.61 |

*ANOVA of model is trending at 0.05<p<0.10

**ANOVA of model is significant at p<0.05

**References**

Harris, A.D., Puts, N.A., and Edden, R.A. (2015). Tissue correction for GABA-edited MRS: Considerations of voxel composition, tissue segmentation, and tissue relaxations. *J Magn Reson Imaging* 42**,** 1431-1440.

Mikkelsen, M., Singh, K.D., Brealy, J.A., Linden, D.E., and Evans, C.J. (2016). Quantification of gamma-aminobutyric acid (GABA) in (1) H MRS volumes composed heterogeneously of grey and white matter. *NMR Biomed* 29**,** 1644-1655.
